# Protective Effect of Total Panax Notoginseng Saponins on Retinal Ganglion Cells of an Optic Nerve Crush Injury Rat Model

**DOI:** 10.1155/2021/4356949

**Published:** 2021-08-04

**Authors:** Huimin Zhong, Huan Yu, Bo Chen, Lei Guo, Xing Xu, Min Jiang, Yisheng Zhong, Jin Qi, Ping Huang

**Affiliations:** ^1^Department of Orthopaedics, Shanghai Key Laboratory for Prevention and Treatment of Bone and Joint Diseases, Shanghai Institute of Traumatology and Orthopaedics, Ruijin Hospital, Shanghai Jiao Tong University School of Medicine, 197 Ruijin 2nd Road, Shanghai 200025, China; ^2^Department of Ophthalmology, Shanghai General Hospital (Shanghai First People's Hospital), National Clinical Research Center for Eye Diseases, Shanghai Key Laboratory of Ocular Fundus Diseases, Shanghai Engineering Center for Visual Science and Photomedicine, Shanghai Engineering Center for Precise Diagnosis and Treatment of Eye Diseases, Shanghai, China; ^3^Department of Ophthalmology, Ruijin Hospital Affiliated Medical School, Shanghai Jiao Tong University, 197 Ruijin Er Road, 200025 Shanghai, China

## Abstract

Irreversible loss of retinal ganglion cells (RGCs) is a common pathological feature of various optic nerve degenerative diseases such as glaucoma and ischemic optic neuropathy. Effective protection of RGCs is the key to successful treatment of these diseases. Total Panax notoginseng saponins (TPNS) are the main active component of Panax notoginseng, which has an inhibitory effect on the apoptosis pathway. This study is aimed at assessing the protective effect of TPNS on RGCs of the optic nerve crush (ONC) model of rats and exploring the underlying mechanisms. The intraperitoneal or intravitreal injection of TPNS was used based on the establishment of the rat ONC model. Fifteen days after the injury, the cell membrane fluorescent probe (Fluoro-Gold) was applied to retrograde RGCs through the superior colliculus and obtain the number of surviving RGCs. TUNEL assay was also used to detect the number and density of RGC apoptosis after the ONC model. The expression and distribution of Bcl-2/Bax, c-Jun/P-c-Jun, and P-JNK in the retina were demonstrated by Western blot analysis. After the intervention of TPNS, the rate of cell survival increased in different retinal regions (*p* < 0.05) and the number of apoptosis cells decreased. Regarding the expression of Bcl-2/Bax, c-Jun/P-c-Jun, and P-JNK-related apoptotic proteins, TPNS can reduce the level of apoptosis and play a role in protecting RGCs (*p* < 0.05). These findings indicate that topical administration of TPNS can inhibit cell apoptosis and promote RGC survival in the crushed optic nerve.

## 1. Introduction

Optic nerve damage is the main cause of irreversible visual impairment. The mechanism is that degenerated axons lead to the gradual death of retinal ganglion cells (RGCs) [1, 2]. Optic neuropathy can also cause color vision changes and abnormal visual fields. Visual information is transmitted from the eyes to the brain through the axons of RGCs. The optic nerve is made up of 1.2 million axons emitted by RGCs [3]. It is conceivable that RGCs has a very close relationship with the optic nerve. Common optic nerve diseases include glaucomatous optic neuropathy, ischemic neuropathy, and inflammatory neuropathy[4].

Panax notoginseng is an ancient Chinese medicine known for its famous therapeutic effects such as promoting blood circulation and removing blood stasis and antidepressant, anti-inflammatory, antitumor, and anti-oxidant properties [5–7]. Panax notoginseng is also an important component of Yunnan Baiyao and many other famous Chinese patent medicines. The main active ingredient saponins can be extracted from caudexes and leaves in Panax notoginseng. The components of total Panax notoginseng saponins (TPNS) include Rb1, Rd, R1, Rg1, Rg2, Rh1, and Rt. In addition, ginsenosides Rg1 and Rb1 which account for the largest proportion are 20% followed by ginsenoside R1 [8, 9]. It has been reported that ginsenoside Rg1 can achieve neuroprotection through antiapoptosis and mitochondrial protein regulation [10–13]. Ginsenoside Rg2 has also been shown to significantly attenuate the neurotoxicity of glutamate-induced PC12 cells [14].

On the other hand, TPNS have an effective effect on anticardiovascular diseases and antidepressant effects, which might be mediated by regulation of Ca^2+^ concentration [6, 15, 16]. Guan et al. exerted ^45^Ca^2+^ trace techniques to prove that TPNS attenuate ^45^Ca^2+^ influx and intracellular free Ca^2+^ level ([Ca^2+^] _i_) [17]. Overloaded Ca^2+^ in cells is the main cause of neuronal death. It is reported that TPNS has been proved to be a calcium channel blocker in neurons, which can block the overload of Ca^2+^ in neurocytes and the production of Ca^2+^-related harmful complexes [18–20].

Thus, TPNS may be a potential therapeutic drug to improve the survival of central neurons and make contribution to clinical treatment of optic nerve damage. In the present study, we analyzed the effect of TPNS on RGC survival and the expression of B-cell lymphoma-2 (Bcl-2), Bax, c-Jun, phosphorylated c-Jun (P-c-Jun), and phosphorylated c-Jun N-terminal kinase (P-JNK), trying to clarify and assess the protective effect of TPNS on RGCs after optic nerve damage in adult rats.

## 2. Materials and Method

### 2.1. Animals

All the described procedures for experimental animals are in compliance with the declarations used in ophthalmology and visual animals issued by ARVO which are all carried out under the guidance of the Animal Protection Committee of Shanghai Jiao Tong University School of Medicine. A total of 104 male adult Sprague-Dawley (SD) rats with an average body weight of 350 g are kept in the animal room of the Shanghai Key Laboratory for Prevention and Treatment of Bone and Joint Diseases, Shanghai Institute of Traumatology and Orthopaedics. SD rats were fed ad libitum and grew up in an environment with a constant temperature of 23°C and a humidity of 60% during the whole experiment. All anesthesia and sacrifices are based on ethical reasons to minimize the suffering of laboratory animals. The number of experimental animals corresponding to their respective experimental procedures is shown in Table 1.

### 2.2. Optic Nerve Crush Model

The experimental rats were anesthetized by intraperitoneal injection of ketamine hydrochloride (25 mg/kg) and fixed on the operating table. Under the operating microscope, the upper fornix conjunctiva of the rat's right eye (operating eye) was cut by about 120° and exposed bluntly to avoid the vortex vein. A 3–4 mm long optic nerve was exposed by opening the meninges of the optic nerve with the sharp tips of forceps, followed by blunt dissection, and then clamped for 1 mm behind the eyeball for 10 seconds with a medium noninvasive vascular clip (about pressure 40 g) [21]. The sham operation control group operated in the same way without optic nerve crush. After the operation, lightly press the cornea and observe under the microscope. If there was no ischemia in the fundus and lens injury, bulbar conjunctiva would be sutured. Then, antibiotic eye drops were given to the eye three times a day for fifteen days and 0.3 ml gentamicin was injected intramuscularly for the first three postoperative days.

### 2.3. Drug Administration and Experiment Grouping

**Figure 1:**
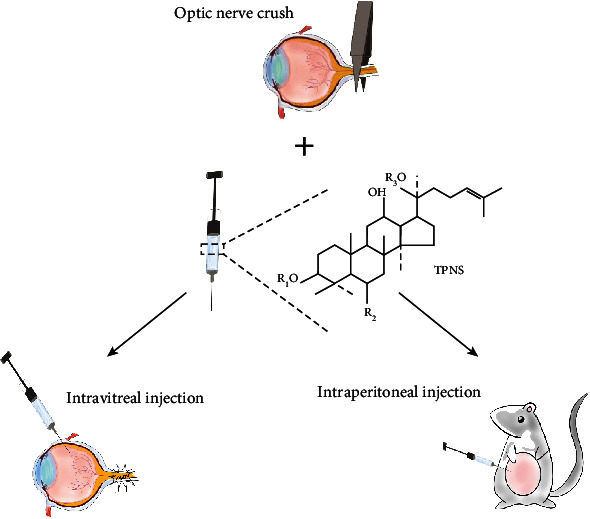
Optic nerve crush model and different ways of administration. Use a noninvasive vascular clip to squeeze 1 mm behind the eyeball to create a rat ONC model. The prepared concentration of TPNS reagent was injected by intravitreal injection and intraperitoneal injection (different injection volumes). The chemical structural formula is the main component of TPNS.

TPNS with a purity of 98% was obtained from Guangxi Wuzhou Pharmaceutical (Group) Co. Ltd. (Guangxi, P.R. China). Briefly, the powder was dissolved in normal saline (approximately 200mg/ml). The experiment was divided into the sham operation control group, optic nerve crush (ONC) group, ONC + TPNS intraperitoneal injection group (ONC + TPNS − IP), and ONC + TPNS intravitreal injection group (ONC + TPNS − IVT). The dose of injection was 150 mg/kg of body weight. The intraperitoneal injection and the intravitreal injection frequency were once every three days. TPNS was injected intravitreally via a stereotactically positioned 32-gauge needle attached to a 10 ml Hamilton syringe. A final volume of 2 *μ*L (the content of TPNS is 4 × 10^−4^ g) was injected via the peripheral temporal retinal site in order to avoid lens injury. After the injection, keep the needle at the injection site for 30 seconds to prevent leakage [22]. Chlortetracycline ophthalmic ointment was applied topically to the treated eye immediately after the intravitreal injection. If vitreous hemorrhage or infection incurred, these rats were excluded instantly (Figure 1).

### 2.4. Retrograde Labeling and Quantification of RGCs

We followed the methods of Huang et al., but the original neuro tracer with less effective was replaced [23]. Four days before the rats were sacrificed, Fluoro-Gold (4%, dissolved in 0.9% saline; Fluorochrome, Denver, CO) was injected into the bilateral superior colliculus to induce the retrograde labeling of RGCs [24]. Briefly, after slicing the skin and bluntly dissociating other tissues, the puncture point was set at the bilateral points 6.4 mm behind the fonticuli anterior, 1.5 mm apart from the midline. The needle of the microinjector was inserted to a depth of 4.0 mm from the skull surface, and 1.5 *μ*L of Fluoro-Gold solution was injected at each point.

**Figure 2:**
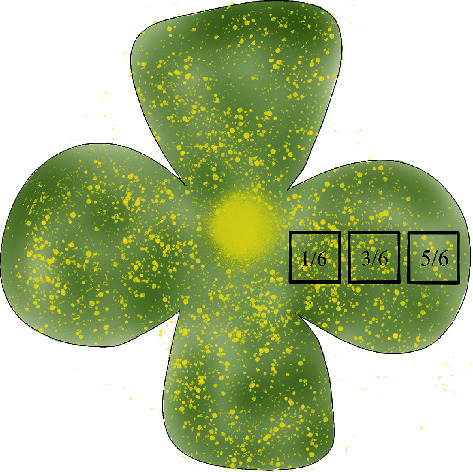
The schematic diagram of the sampling position for RGC quantification in the retina. The retinal neuroepithelium layers were cut into a cross and spread on the glass slide. Taking the optic papilla as the center, retina areas have been divided into 1/6, 3/6, and 5/6.

Four days after the labeling, the rat heart was perfused and fixed by normal saline and paraformaldehyde and then the eyeballs could be taken. After fixation in 4% paraformaldehyde for 1 hour, the retinal neuroepithelium layers were dissociated and completely mounted on slides. Images of labeled RGCs were captured immediately by a Zeiss Imager M1 laser-scanning microscope (Carl Zeiss Microscopy) under dark conditions. The quantification of RGCs was counted in areas of approximately the same distances of 1/6, 3/6, and 5/6 retinal radii from the optic disk (Figure 2), using a digital image analysis system (Image-Pro Plus version 6.0; Media Cybernetics, Silver Spring, MD). The average of the RGC density values under the same eccentricity can be considered as the mean density of RGCs for the certain position in each retina.

### 2.5. TUNEL Assay

First of all, rats were still fixed by cardiac perfusion after anesthesia. The right eye was removed and fixed in 4% paraformaldehyde overnight. Then, the optic cup was separated with the vitreous body and the sucrose solution was exerted for gradient dehydration. In the next step, the processed eyecups were embedded and frozen in an optimal cutting temperature compound (Sakura Finetek, Torrance, CA). A sagittal section of the retina was performed with a thickness of 7 *μ*m. The slices were assayed with a terminal deoxynucleotidyl transferase- (TdT-) mediated deoxyuridine triphosphate (dUTP) nick end labeling (TUNEL) method, using an In Situ Cell Death Detection Kit (Roche Diagnostics Corporation, Indianapolis, IN). In brief, the air-dried slices were permeated with cold 0.25% Triton X-100 solution (Sigma–Aldrich) for 30 minutes at room temperature and then incubated with 100 *μ*L of the TUNEL mixture system in a humidified atmosphere at 37°C for 1 hour. The processed slices were mounted with a prolong antifade medium (Life Technologies) combined with DAPI for five minutes is the last step before capturing. Images were captured immediately by a Zeiss Imager M1 laser-scanning microscope (Carl Zeiss Microscopy), and the number and density of TUNEL-positive RGC apoptosis in each group were analyzed using a digital image analysis system (Image-Pro Plus version 6.0; Media Cybernetics, Silver Spring, MD).

### 2.6. Western Blot Analysis

Retinas were collected and immersed in radioimmunoprecipitation assay buffer (RIPA; Sigma–Aldrich) with a protease inhibitor cocktail (Complete Protease Inhibitor Cocktail; Roche Applied Science, Penzberg, Germany) and phosphatase inhibitor (Sigma–Aldrich). The lysis process was entirely performed on ice, and then, lysates were fully homogenized (frequency 55 Hz, 60 seconds). The supernatant after centrifugation was quantified with a BCA kit (Bicinchoninic Acid Kit; Sigma–Aldrich) for the protein concentration of the retinal homogenate. Equal amounts of protein samples were separated with 12.5% SDS-PAGE in a Mini-PROTEAN 3 Electrophoresis System (Bio-Rad, Hercules, CA) and then electroblotted to polyvinylidene fluoride membranes (PVDF) (Merck Millipore, Billerica, MA). After being blocked with rapid block buff at room temperature for 15–20 minutes, the membranes were incubated with rabbit polyclonal anti-BAX (1 : 1000 dilution, ab-53154; Abcam), rabbit polyclonal anti-BCl-2 (1 : 1000 dilution, ab-196495; Abcam), mouse monoclonal anti-JNK (1 : 1000 dilution, sc-7345; Santa Cruz Biotechnology), mouse monoclonal anti-p-JNK (1 : 500 dilution, sc-6254; Santa Cruz Biotechnology), mouse monoclonal anti-c-Jun (1 : 500 dilution, sc-74543; Santa Cruz Biotechnology), mouse monoclonal anti-p-c-Jun (1 : 500 dilution, sc-822; Santa Cruz Biotechnology), and mouse monoclonal anti-*β*-actin (1 : 1000 dilution, #3700; Cell Signaling Technology Inc.) primary antibodies at 4°C overnight.

After washing with Tris-buffered saline with Tween sufficiently, the membranes then were incubated with horseradish peroxidase-conjugated goat anti-rabbit (1 : 2000 dilution; Abcam) or goat anti-mouse (1 : 2000 dilution; Abcam) secondary antibodies at room temperature for 1 h. The protein bands on the membranes were detected by using a chemifluorescence reagent (Beyotime, Shanghai, CN) in an ImageQuant LAS 4000 mini system (GE Healthcare Bio-Sciences, Piscataway, NJ). Images were analyzed quantitatively by an image analysis system (Image-Pro Plus Version 6.0; Media Cybernetics, Silver Spring, MD).

### 2.7. Statistical Analysis

The statistical data were expressed as mean ± standard deviation. All data statistics were processed with SPSS 13.0 software (IBM SPSS Statistics version 19.0; IBM, Armonk, NY) and GraphPad Prism 9.0. The Kruskal-Wallis rank sum test and Bonferroni were used to assess the statistical significances, which were determined as *p* < 0.05.

## 3. Results

### 3.1. TPNS Improved RGC Survival under ONC

**Figure 3:**
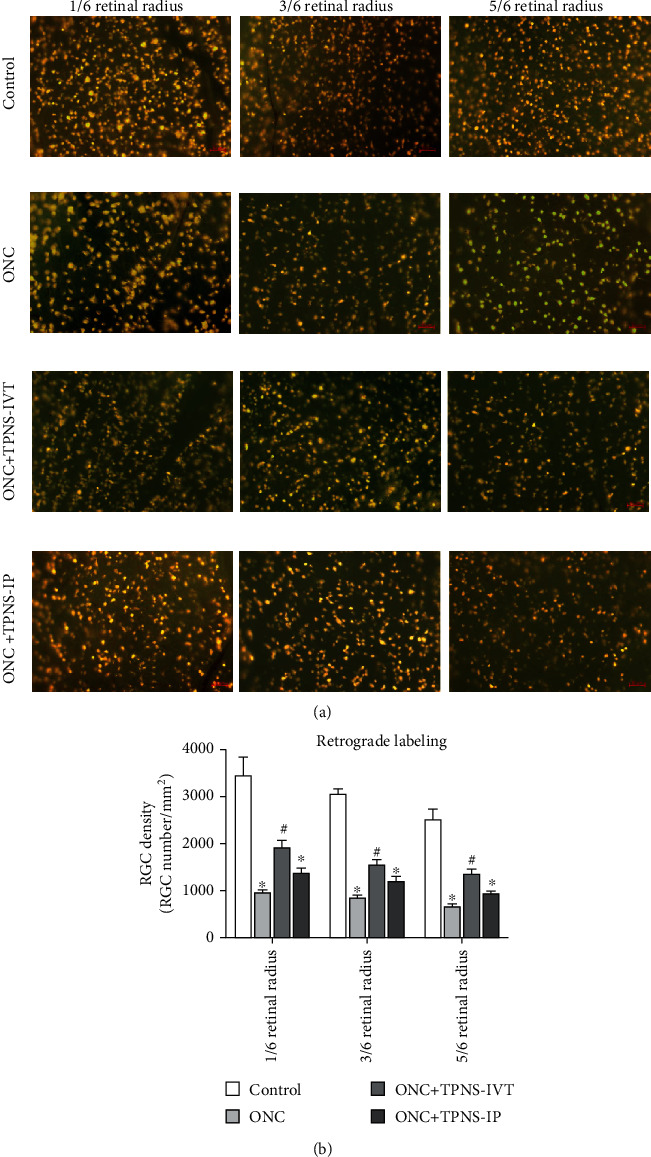
TPNS promotes RGC survival after optic nerve injury. Retinal images were taken using a fluorescent microscope. (a) Representative pictures of Fluoro-Gold retrograde labeling of RGCs in retinal flatmounts from the control, ONC, ONC + TPNS − IVT, and ONC + TPNS − IP groups, as marked. Scale bar = 50 *μ*m. (b) Average RGC survival summed across 6 of each sampling region averaged by the unit area for each experimental group (*n* = 6; mean ± SD; *p* values are indicated at the top of bars, ^∗^*p* < 0.05 versus the control group, and ^#^*p* < 0.05ONC + TPNS − IVT group versus the ONC group).

After fifteen days of ONC accompanied by regular drug administration, bilateral superior colliculus retrograde Fluoro-Gold labeling and RGC counting were performed (Figure 3(a)). In the sham operation control group, a higher RGC density was located in the retina near the optic nerve head, while a lower density was observed in the peripheral retina (3457 ± 350/mm^2^ in a 1/6 retinal radius, 3061 ± 117/mm^2^ in a 3/6 retinal radius, and 2527 ± 197/mm^2^ in a 5/6 retinal radius). After fifteen days of optic nerve crush, there were significant differences in the RGC density of corresponding retinal areas among the sham operation control group, ONC group, ONC + TPNS − IP group, and ONC + TPNS − IVT group (Kruskal-Wallis: *p* < 0.05 for a 1/6 retinal radius, 3/6 retinal radius, and 5/6 retinal radius, *n* = 6). Comparing with the sham operation control group, the RGC density decreased significantly in all three retinal positions in the ONC group (969 ± 12/mm^2^ in a 1/6 retinal radius, 842 ± 27/mm^2^ in a 3/6 retinal radius, and 653 ± 38/mm^2^ in a 5/6 retinal radius), ONC + TPNS − IVT group (1918 ± 151/mm^2^ in a 1/6 retinal radius, 1542 ± 90/mm^2^ in a 3/6 retinal radius, and 1331 ± 106/mm^2^ in a 5/6 retinal radius), and ONC + TPNS − IP group (1373 ± 86/mm^2^ in a 1/6 retinal radius, 1184 ± 107/mm^2^ in a 3/6 retinal radius, and 924 ± 68/mm^2^ in a 5/6 retinal radius). The RGC density in the three positions of the retina after ONC were significantly reduced (*p* < 0.05). Meanwhile, the RGC density in each retinal positions in the ONC + TPNS − IVT group performed positive significantly higher than that in the ONC group while that in the ONC + TPNS − IP group did not (*p* < 0.05 in all regions) (Figure 3(b)). The results presented that TPNS effectively improved the survival number of RGCs in the ONC rats. Furthermore, intravitreal injection was better from the perspective of drug delivery.

### 3.2. TPNS Attenuated RGC Apoptosis under ONC

**Figure 4:**
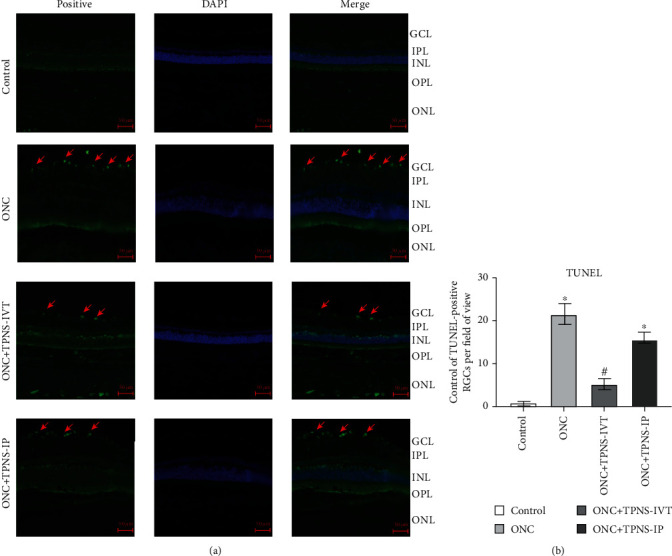
Apoptosis of RGCs fifteen days after ONC in each group. (a) The green marked area pointed by red arrows represents the apoptosis cellular staining of RGCs/field. Magnification: 200; scale bar: 50 *μ*m. Magnified images of ON head region. Scale bar: 50 *μ*m. GCL: ganglion cell layer; INL: inner nuclear layer; IPL: inner plexiform layer; ONL: outer nuclear layer; OPL: outer plexiform layer. (b) There was a significant difference in the number of TUNEL-positive optic ganglion cells (RGCs) fifteen days after nerve injury compared with the control group. Fifteen days after nerve injury, the number of RGCs with positive TUNEL staining in a single visual field was significantly reduced in the ONC + TPNS − IVT group compared with the ONC group (*n* = 5, ^∗^*p* < 0.05 versus the control group, and ^#^*p* < 0.05 versus the ONC group).

To validate whether the previous retrograde results coincided with those in retina cell apoptosis, TUNEL assay was performed immediately to evaluate the therapeutic effect of TPNS on RGC apoptosis after fifteen days of ONC. There were differences in the number of apoptotic RGCs among the sham operation control group, ONC group, ONC + TPNS-IVT group and ONC + TPNS-IP group through immunostaining. Comparing with the sham operation control group, the apoptotic RGCs all appeared in the ONC group, ONC + TPNS-IP group and ONC + TPNS-IVT group. What's more, less cellular staining of apoptosis cells in the ONC + TPNS-IVT group seems to be found through immunostaining when compared with ONC + TPNS-IP group and ONC groups. As a result, we count the number of TUNEL-stained cells in a single field of view (n = 5, P <0.05). At fifteen after injury of optic nerve, Apoptosis cells were observed in frozen sections of rat retinal. TUNEL staining was used to detect the apoptosis of RGCs after different treatments. It showed that obviously, a significant number change was observed in the ONC group and the apoptotic number of RGCs was greatly reduced after intravitreal injection of TPNS drugs. The results indicated that TPNS may have a potential antiapoptotic effect on RGCs in ONC. (Figures 4(a) and 4(b)).

### 3.3. Effect of TPNS on Apoptotic-Related Protein Expression in the Retina under ONC

**Figure 5:**
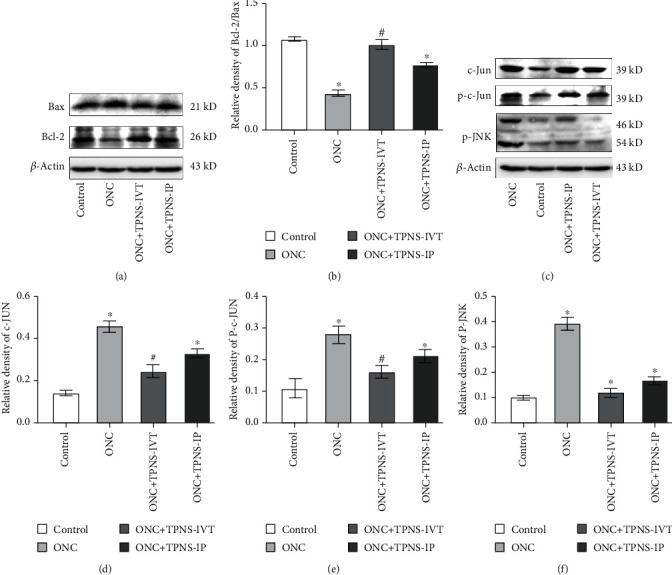
Activity of the intrinsic apoptotic pathway was suppressed by TPNS treatment in ONC rats. (a) Quantitative representative Western blotting bands of retinal Bcl-2, Bax, and internal controlling *β*-actin protein expression of different groups. (b) Semiquantitative analysis of Western blotting grey values for Bcl-2/Bax (*n* = 6, ^∗^*p* < 0.05 versus the control group, and ^#^*p* < 0.05 versus the ONC group). (c) Quantitative representative Western blotting bands of retinal P-JNK, c-Jun, P-c-Jun, and internal controlling *β*-actin protein expression of different groups. (d–f) Semiquantitative analysis of Western blotting grey values for P-JNK, c-Jun, and P-c-Jun (*n* = 6, ^∗^*p* < 0.05 versus the control group, and ^#^*p* < 0.05 versus the ONC group).

After fifteen days of ONC, Western blotting was performed immediately to evaluate the apoptotic-related protein expression in the retina. The results showed that the relative ratio of Bcl-2/Bax protein expression (Bcl-2/Bax) in the ONC group was significantly lower than that in the sham operation control group (*p* < 0.05). The experiment detected that Bcl-2/Bax was markedly reversed by TPNS treatment. Bcl-2/Bax in the ONC + TPNS − IVT group was significantly higher than that in the injury group (*p* < 0.05) (Figures 5(a) and 5(b)).

Similarly, c-Jun/P-c-Jun and P-JNK also play a facilitating role in the apoptosis pathway. Western blotting showed the similar tendencies of variations that the retinal protein expression of c-Jun/P-c-Jun and P-JNK has in each group (Kruskal-Wallis: *p* < 0.05, *n* = 6). After intravitreal and intraperitoneal injection of TPNS, c-Jun protein expression was decreased by 46.0% and 27.4%, respectively. In the same way, P-c-Jun and P-JNK protein expression also has obviously change compared with that of the ONC group, which shows that that of the ONC + TPNS − IVT group is reduced by 42.1% and 70.2%, respectively (*p* < 0.05 for P-c-Jun and P-JNK). From the relevant data, we can clearly find that intravitreal injection has a better trend than intraperitoneal injection because intraperitoneal injection is not significantly different from ONC (Figures 5(c)–5(f)). This trend and retrograde data perfectly corroborated each other.

## 4. Discussion

There are many reports on the protective effect of the main components of TPNS on neurons in the central nervous system diseases and improvement of mental function. In Alzheimer's disease characterized by neuro loss and cognitive impairment, TPNS can protect nerves by targeting circRNA expression, regulating brain metabolism-related pathways, regulating mitochondrial proteins, and inhibiting tau protein phosphorylation [13, 25–27]. On the other hand, TPNS can also protect neurons through antiapoptotic and anti-inflammatory pathways to exert antidepression, anxiety, and cognitive deficits and other mental symptoms [28, 29]. Additionally, physiological Ca ^2+^ signals are essential for cell function and survival. Overload Ca ^2+^ or disturbance of intracellular Ca ^2+^ partitions can activate or enhance the mechanism leading to cell death or apoptosis [30]. The inhibitory effect of panax notoginseng on the increase of [Ca^2+^] _i_ may be the basis for protection against neuronal cell damage induced by excitatory amino acids or neurotoxins. The neurotoxic effect of N-methyl-D-aspartate (NMDA) mediated by calcium influx of nerve cells leads to apoptosis and cell death [6, 31, 32]. Thus, calcium channel blockade is considered to be reasonable by inducing vasodilation to improve the local blood flow of ischemic tissue, thereby achieving neuroprotection [33]. TPNS can inhibit Ca^2+^ influx in cardiovascular diseases and regulate intracellular Ca^2+^ concentration in depression to achieve antidepressant effects [6, 17]. In the same way, TPNS, which has a calcium channel blocking effect, can also protect nerve cells by inhibiting the influx of calcium ions.

So far, even though traditional Chinese medicine has been extensively studied, the mechanism of traditional Chinese medicine is still complicated and needs to be resolved. The retina, which is part of the central nervous system, is an ideal research object. Retinal neurons such as RGCs do not regenerate so that strategies aimed at protecting and regenerating these cells are currently a focus topic. However, TPNS has limited research on ocular diseases like optic nerve damage. In this study, TPNS was shown to protect the optic nerve in terms of antiapoptosis and reducing the loss of RGCs.

In our model, a 10 second crush model was used to reproducibly injure the optic nerve with very little risk to the blood flow and ophthalmic artery. Tan et al. proved that compared with mild (5 seconds) and severe (30 seconds) optic nerve crush injury models, the moderate (10 seconds) optic nerve crush injury model was more suitable for the study of optic nerve injury and regeneration [21]. Notably, our research chose fifteen days as the time point because it had been reported that approximately 50% of RGCs start the apoptosis program fifteen days after injury and are maintained at least 4 months [34].

Previous studies demonstrated that optical coherence tomography showed that the RGC layer gradually decreased after optic neuropathy [4]. In our study, we exerted retrograde labeling to detect survival whereas TUNEL assay to test apoptosis. Obviously, the survival rate of RGCs that could be retrogradely labeled in the ONC group was significantly lower than that in the sham operation group (control group) at fifteen days postoperatively. After we used intravitreal injection and intraperitoneal injection to regularly inject drugs after surgery, the number of RGCs surviving increased significantly. TUNEL assay has been designed to detect apoptotic cells that undergo extensive DNA degradation during the late stages of apoptosis [35]. Therefore, TUNEL-positive cells mean apoptotic RGCs. In our results, no apoptotic cells were detected in the control group while more TUNEL-positive cells were labeled in the ONC group. Although apoptotic RGCs could be detected in the TPNS injection group, the number was significantly lower than that in the ONC postoperative group. The cell number of cellular staining of the ONC + TPNS − IVT group was slightly less than that of the ONC + TPNS − IP group. On the other hand, the local injection was more advantageous for the protective effect of TPNS on RGCs. (Figures 4(a) and 4(b)).

Optic nerve damage lies in the loss and/or degeneration of neuronal axons followed by loss of cells that induce optic nerve atrophy by RGC apoptosis [35]. Neuronal apoptosis is a very complex cascade process involving multiple processes, and many apoptotic pathways feature in it [36].

There are many proteins related to apoptosis, such as representative antiapoptotic proteins Bcl-2 or proapoptotic proteins such as Bax, P-JNK, C-Jun, and P-C-Jun. The ratio of Bcl-2/Bax reflects the activation of the apoptosis program [37–40]. In the case of the apoptotic pathway of RGCs in the ONC process, the increase in the Bcl-2 level and the decrease in the Bax level appear to indicate that cells are more resistant to apoptosis (Figures 5(a) and 5(b)). In our study, the semiquantitative analysis of Western blotting has proved that the decreased relative ratio of Bcl-2/Bax protein expression in ONC rats was both significantly reversed by TPNS treatment. The results further demonstrated the antiapoptotic effect of TPNS through regulation of the apoptotic pathway in optic nerve damage diseases.

Similarly, our results illustrated that the retinal protein expression of P-JNK, c-Jun, and p-c-Jun increased generally in the ONC rats, which could explain the discovery that the apoptosis level in the ONC rats activated obviously in comparison with that in the control. (Figures 5(c)–5(f)). C-Jun is a representative member of the immediate early genes responding and adapting to a variety of stimuli and also participating in the transcription process of the effector enzymes of the signal transmission system [41]. P-JNK acts as key proteins involved in the cells' stress responses [42]. NMDA is involved in calcium influx of nerve cells and induced excitotoxicity, thereby increasing the level of P-JNK protein [43]. P-JNK levels decreased after TPNS intervention thus reducing neurotoxicity and protecting nerves. Elevated levels of c-Jun can cause neuronal cell apoptosis, and phosphorylated c-Jun can cause cell death by upregulating genes that induce cell death [44]. Optic nerve damage makes c-Jun/P-C-Jun express rapidly, and overexpression of c-Jun/P-C-Jun activation can cause neuronal apoptosis. In addition, P-JNK, c-Jun, and p-c-Jun are also closely related to oxidative stress and inflammation pathways [45, 46]. Therefore, TPNS has inhibitory effects on cell apoptosis pathway induced by optic nerve injury mechanistically.

What is more, intravitreal medication seems to be better than intraperitoneal injection in either TUNEL or the apoptosis pathway although both intravitreal injection and intraperitoneal injection have an effect on neuroprotection. The reason of the conclusion was considered that the effect of the local action was better than the systemic one. Experiments had proved that direct ocular applications could achieve the biological effects of the posterior segment of the eye, so as to further understand the diffusion and transport in the optic nerve [47]. Meanwhile, Ercan et al. had found that intraperitoneal injection had diminished experiment effect compared with intravitreal injection from their results [48].

On the other hand, intravitreal injection may protect the eye more efficiently without undesirable systemic influence in spite of the same concentration of two injection methods. For relatively localized eyes, local administration of TPNS at a much lower dose provides greater advantages for the treatment of optic nerve injury, because systemic intraperitoneal administration is likely to have unknown effects on other organs. Nevertheless, it is still necessary to clarify the detailed pharmacokinetic characteristics of TPNS in the eye [49]. The operation of intravitreal injection is most susceptible to infection and endophthalmitis. At present, the injection of antibiotics into the vitreous cavity is still controversial and may even increase the risk of infection so we still exert the prevention of systemic and local antibiotics. Strict and precise control of aseptic operation is the most important preventive measure [50, 51]. On this regard, it could be useful to develop in the near future a biodegradable deliver system to inject intravitreally the TPNS, avoiding a multiple treatment [52].

In a word, we illustrated that TPNS can inhibit cell apoptosis and promote RGC survival in the crushed optic nerve, which implying a potential neuroprotective therapy for neurological disorders. In the comparison of different administration methods, intravitreal injection of TPNS under absolutely aseptic conditions is a trend in the treatment of optic nerve injury.

## 5. Conclusions

Collectively, our study shows that TPNS can reduce the loss of RGCs through the antiapoptotic pathway to achieve the function of treating nerve injury. However, how TPNS leads to apoptosis in optic nerve injury and the research on the axon function and phenotypic of neurons need to be further studied. TPNS may be a potential and relatively cheap drug targeting neuroprotection. In the future, the protective retinal role of TPNS in retinal pathology should be focused on intraocular pharmacokinetics and new intravitreal formulations.

## Figures and Tables

**Table 1:** Number of rats used in the respective experimental procedures.

Procedure	Number
Retrograde labeling	32
Western blot	32
TUNEL assay	40
Total	104

## Data Availability

The data used to support the findings of this study are available from the corresponding author upon request.
